# Candidate composite biomarker to inform drug treatments for diabetic kidney disease

**DOI:** 10.3389/fmed.2023.1271407

**Published:** 2023-11-01

**Authors:** Roger D. Jones, Seyum Abebe, Veronica Distefano, Gert Mayer, Irene Poli, Claudio Silvestri, Debora Slanzi

**Affiliations:** ^1^European Centre for Living Technology, Ca' Foscari University of Venice, Venice, Italy; ^2^Department of Biology, University of North Carolina at Chapel Hill, Chapel Hill, NC, United States; ^3^Systems Engineering and Research Center, Stevens Institute of Technology, Hoboken, NJ, United States; ^4^Department of Economic Sciences, Università del Salento, Salento, Italy; ^5^Internal Medicine IV, Medical University Innsbruck, Innsbruck, Austria; ^6^Department of Environmental Sciences, Informatics and Statistics, Ca' Foscari University of Venice, Venice, Italy; ^7^Department of Management, Ca' Foscari University of Venice, Venice, Italy

**Keywords:** precision medicine, diabetic kidney disease, biomarkers, RASi, SGLT2i, MCRa, clinical data

## Abstract

**Introduction:**

Current guidelines recommend renin angiotensin system inhibitors (RASi) as key components of treatment of diabetic kidney disease (DKD). Additional options include sodium-glucose cotransporter-2 inhibitors (SGLT2i), glucagon-like peptide 1 receptor agonists (GLP1a), and mineralocorticoid receptor antagonists (MCRa). The identification of the optimum drug combination for an individual is difficult because of the inter-, and longitudinal intra-individual heterogeneity of response to therapy.

**Results:**

Using data from a large observational study (PROVALID), we identified a set of parameters that can be combined into a meaningful composite biomarker that appears to be able to identify which of the various treatment options is clinically beneficial for an individual. It uses machine-earning techniques to estimate under what conditions a treatment of RASi plus an additional treatment is different from the treatment with RASi alone. The measure of difference is the annual percent change (ΔeGFR) in the estimated glomerular filtration rate (ΔeGFR). The 1eGFR is estimated for both the RASi-alone treatment and the add-on treatment.

**Discussion:**

Higher estimated increase of eGFR for add-on patients compared with RASi-alone patients indicates that prognosis may be improved with the add-on treatment. The personalized biomarker value thus identifies which patients may benefit from the additional treatment.

## 1. Introduction

Cross sectional inter- and longitudinal intra-individual heterogeneity in progression and response to therapy is a common feature of many chronic and age-related diseases. The current state-of-the-art guideline-backed clinical practice relies on studies in large cohorts and does not take individual variability into account. Precision/personalized/stratified medicine attempts to identify the individual prognosis and targeted treatment at the right time for the right patient, or at least for smaller and more homogeneous groups (1–3). Implementation requires adaptations in research as well as in clinical approaches. As an example, patients with diabetes mellitus type 2 and kidney disease (diabetic kidney disease; DKD) are currently categorized by two biomarkers, the estimated glomerular filtration rate (eGFR), a measure of the kidneys ability to excrete waste products and the amount of pathologically increased excretion of albumin in the urine (4). Even though each of these alterations reflects a distinct pathology with impact on prognosis (5–7) and therapy is adjusted accordingly, heterogeneity in response persists. If we increase the data space for deeper phenotyping (including e.g., genetics, family and personal history, lifestyle, environment, demographics, routine laboratory parameters or even Omics profiling studies), we must use higher-resolution statistics to extract usable information. Systems biology (8) and advanced data-mining techniques are required to (1) improve phenotyping, (2) predict the future state (prognosis) of the individual, and (3) identify the most effective spectrum of drugs to intervene. The design of clinical validation trials also needs adjustment to the individual or small cluster level (9). Increased molecular resolution of pathophysiology as well as drug mode of action will also improve our understanding of diseases and support the process of drug discovery (10). As an example, the hormone angiotensin II increases blood pressure and prolonged hypertension drives DKD. Renin angiotensin system inhibitors (RASi), such as angiotensin converting enzyme inhibitors (ACEis) and angiotensin II receptor blockers (ARBs), block the formation and action of angiotensin II and lower systemic blood pressure. Interestingly, when compared with other antihypertensive agents, ACEis and ARBs stabilize kidney function at the same level of achieved blood pressure better than conventional antihypertensive therapy (11). This suggests that angiotensin II also operates in other processes (10, 12). Indeed, the angiotensin receptor and other G-protein coupled receptors can trigger distinct multiple downstream responses that depend on the cellular environment (13–15) and thereby may lead to heterogeneous disease progression and effect of therapy.

In this study, we focused on the identification of a biomarker panel to support precision drug treatment in DKD. We used data from a subgroup of patients included in the PROVALID study (16–19), a longitudinal prospective observational study in patients with type 2 diabetes. Information on eGFR and therapy as well as many other biomarkers was available on an annual basis. Patients with controlled kidney disease (CD) were characterized by an annual decrease of eGFR not exceeding 5%, while eGFR dropped more than 10% in uncontrolled DKD (UCD). A 1-year follow-up period is consistent with international guideline recommendations. The 1-year follow-up minimizes the effect of longitudinal intra-individual heterogeneity in treatment response and our threshold of 10% decrease for identification of uncontrolled disease still represents a change in eGFR not expected to occur spontaneously (18). All patients were continuously treated with a RASi. In some individuals, one other agent supposed to beneficially affect DKD [glucagon-like peptide 1 agonist (GLP1a), mineralocorticoid receptor antagonist (MCRa) or a sodium-glucose cotransporter 2 inhibitor (SGLT2i)], was added on top of RASi therapy in the 1-year follow up period. We set out to define a biomarker panel that supports clinicians to decide if a patient, who is currently treated with an ACEi or ARB-only should remain on this regimen as CD is expected or be changed to a drug combination to improve outcome in case of UCD prognosis. To answer this question at the most basic level, we use data to construct a model Δ_*R*_ that predicts the future value of the change (ΔeGFR) in eGFR between baseline and the next follow-up visit in RASi-only treated patients. Next, we apply the model to patients taking one of the other three combination drug therapies. If the added treatment has no effect, we expect the RASi-only model to predict the outcome accurately. If, however, the other treatment has an effect beyond that of the effect of RASi only, the model will not be a good predictor. Next, we developed models, Δ_*G*_, Δ_*M*_, and Δ_*S*_, to predict ΔeGFR for each individual drug of interest, GLP1a, MCRa, and SGLT2i, respectively, when added on top of RASi. If a new patient thus presents on RASi only therapy and that patient's value for Δ_*R*_ is measured and calculated, the physician can decide if the individual should stay on RASi only or not. In those with a negative prognosis on RASi alone, the change in ΔeGFR expected under different combination therapies can be estimated, and the best therapy is selected. The process described will become part of a toolbox that supports clinicians treating patients with DKD. We therefore recognized a number of practical constraints on biomarker selection and on the prediction model. Clinical tests can be time-consuming and expensive, and therefore, the selection of variables should ideally be restricted to a small number of readily available and inexpensive parameters, if possible. In addition, they should be familiar and explainable to clinicians and ideally be linked to relevant biological processes. Moreover, as many patient tests will pass through the toolbox, the models should be computationally efficient.

## 2. Approach

Data on patients with DKD used in this study were obtained in an extensive data-collection effort, the PROVALID (PROspective cohort study in patients with type 2 diabetes mellitus for VALIDation of biomarkers) study (16–19). Here, 4,000 patients were recruited at the primary level of healthcare in Austria, Hungary, Netherlands, Poland, and Scotland. The patients visited their physicians annually as part of standard clinical practice and were followed for at least 4 years. Information on patient history, physical status, laboratory measurements, medication, and renal and cardiovascular events were collected as well as urine and plasma for measurement of biomarkers. We used the Modification of Diet in Renal Disease Study equation (MDRD) formula for the calculation of ΔeGFR (20). Only individuals with eGFR values between 30 and 90 ml/min/1.73 m^2^ were included. The prevention of progression of DKD (defined as a loss of eGFR) is most efficient in early disease. Therefore, we excluded individuals with advanced stages (i.e., an eGFR <30 ml/min/1.73 m^2^). On the contrary, hyperfiltration with elevated eGFR is atypical early feature of DKD. The pathophysiology of induction and resolution of hyperfiltration is not completely clear but may be different from progression thereafter. Hence, we decided to set the upper boundary of eGFR for inclusion to 90 ml/min/1.73 m^2^. Baseline characteristics and medication for all participants per group are presented in the Supplementary material. For this analysis, patients with the following treatment regimen were selected:

1. RASi as the only drug treatment during a 1-year follow-up period (RASi only). For this group, the same patient could contribute multiple annual sequences, and we aimed for a equal distribution of CD and UCD.
2. RASi during a 1-year follow-up period with addition of a glucagon-like peptide 1 agonist added after baseline (RASi+GLP1a).
3. RASi during a 1-year follow-up period with addition of a mineralocorticoid receptor antagonist added after baseline (RASi+MCRa).
4. RASi during a 1-year follow-up period with addition of a sodium-glucose cotransporter 2 inhibitor added after baseline (RASi+SGLT2i).

The data presented several challenges to modeling:

1. The number of visits per group was small (approximately 100) with the exception of the RASi only group. As a consequence, data-hungry multi-layer perceptrons, for instance, are not adequate for non-linear modeling of this particular sparse data. Machine-learning techniques that can efficiently extract information from small amounts of data were required.
2. The evolution of ΔeGFR, as seen in PROVALID data, can change significantly within a period of 1 year, which is the measurement interval (21). This requires modeling techniques that can change predictions discontinuously over a period of 1 year. For this, we used recurrent neural networks that handle discontinuities in data.
3. Physician visits by the same patient are correlated with each other leading to co-linearity issues in the building all the models in this study. As a consequence, we removed collinearity by preprocessing the data with the partial least squares (PLS) algorithm, which is designed to minimize the effects of collinearity.
4. The results should be biologically interpretable, which is a ubiquitous concern of statistical modeling and will be a constraint on variable panel selection. As a consequence, we relied on data, that are available in daily clinical routine (e.g., blood pressure urinary albumin excretion). These variables were complemented urinary or plasma proteins that were identified to be part of DKD pathophysiology and molecular drug mode of action by bioinformatical analysis. The complete list of parameters available is given in Supplementary Table 1.

Variables were selected from the complete PROVALID data set and a subset of PROVALID as suggested by experts in (22) (**Table2**). Only continuous but not discrete and binary variables were included. We used the partial least squares algorithm to determine the baseline variables that were most accurately correlated with ΔeGFR in the complete PROVALID set. We did the same for the subset of variables in the expert selection, and we combined the most important variables from each calculation into a single data set and performed the PLS calculation on this combined set, selecting the most important variables (see the nine variables selected in **Table 2**). We then used a hybrid machine-learning technique (see Appendix) to predict ΔeGFR for the group of patients treated with RASi only. The model (PLSNN) is a combination of partial least squares (PLS) and normalized radial basis function neural network (NN). The prediction of ΔeGFR for the RASi-only set of patients is the composite biomarker Δ_*R*_, which will allow the physician to estimate ΔeGFR within the next year (and thus the state of CD or UCD) in case the patient remains on RASi only. In case UCD is predicted, similar models for the other treatment option groups will enable the clinician to select the best option to maintain kidney function.

## 3. Results

**Figure 1:**
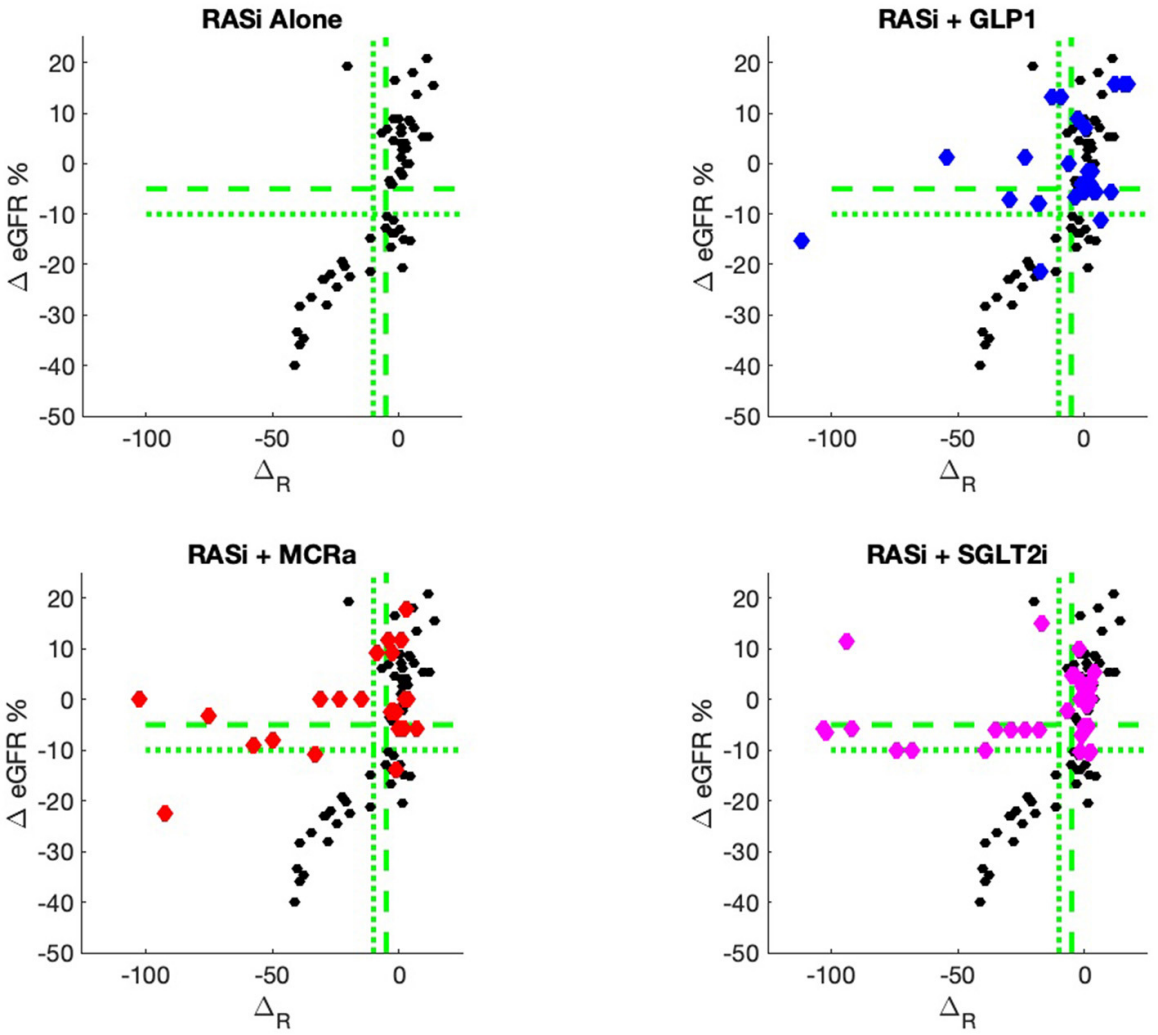
PROVALID data output ΔeGFR as a function of the composite biomarker Δ_*R*_. Here, Δ_*R*_ is the predicted output for the RASi Alone model. The black markers represent data for RASi only, and the colored markers represent data for the add-on drugs. If the colored markers have greater values for Δ*e*GFR than the black markers, then possible benefit from the add-on drug may be indicated. The green dashed line indicates values for Δ*e*GFR and Δ_*R*_ that are equal to −5%. The dotted green lines are for values of −10%. These values are often used as markers for controlled and uncontrolled DKD.

**Figure 2:**
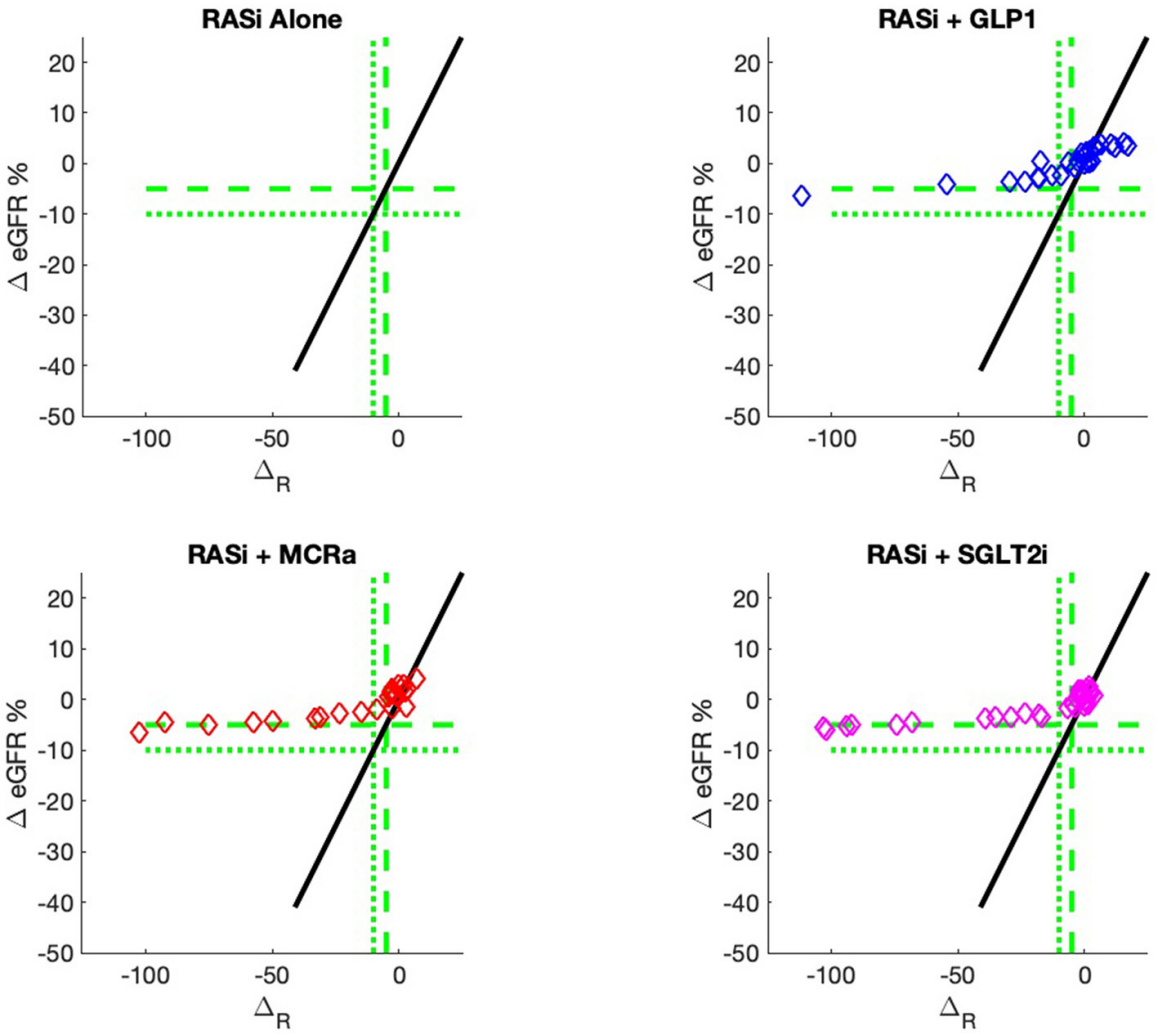
Model outputs for RASi only (black) and RASi plus add-on drugs (colored) vs. the composite biomarker Δ_*R*_. The add-on outputs are the expectation of ΔeGFR, just as the case for RASi-only. These outputs are designated Δ_*G*_, Δ_*M*_, and Δ_*S*_ for GLP1a, MCRa, and SGLT2i, respectively. The vertical distance between the add-on model and the RASi-only model is the expected increase/decrease in ΔeGFR for a patient with composite biomarker Δ_*R*_ and administration of the add-on drug. The use of models allows direct comparison of the add-on drug outcome to the RASi-only outcome. The models can be calculated for any values of input biomarkers, while the actual data of Figure 1 only contains output data for a discrete set of sample inputs and, thus, cannot give comparisons for all values of input biomarkers.

The nine continuous input variables as given by experts in (22, 23) are displayed in Table 1. Discrete variables were not included in this study because the modeling process used here is restricted to continuous inputs. The highest PLS-ranked continuous variables from the PROVALID set and the expert data set were combined, re-ranked by PLS, and the consensus file is displayed in Table 2. The Figure 1 upper left panel shows the relation between Δ_*R*_ and ΔeGFR in the RASi-only treatment group (black dots). A decrease of 10% or more of Δ_*R*_ characterizes patients with UCD (drop in eGFR more than 10%, dotted green lines) and higher values of Δ_*R*_ those with CD (dashed green lines). The upper right and the lower panels show the observations for the add-on treatments (colored dots) vs. the RASi-only model prediction. The RASi-only model predicted well for individuals with CD regardless of the type of add-on therapy. On the other hand, patients in whom the RASi-only model predicted UCD clearly showed a different outcome when drugs were added, with most moving to the CD population. Of note, the lower the Δ_*R*_ is, the larger is the effect of the add-on drug. This supports the hypothesis that addressing a different pathophysiology via a specific drug mode of action is beneficial in patients with a disease trajectory unresponsive to RASi therapy alone. As Δ_*R*_ developed for RASi to predict ΔeGFR obviously was not accurate in the add-on therapy groups, we developed models for the individual groups using the same variables. Prediction models were developed in each of the four treatment populations, and the variable ranking regarding informational contribution within each group (providing insight into pathophysiology) is displayed in Table 3. The models were tested on each of the four treatment populations. The predictions of ΔeGFR and sensitivity as well as specificity and accuracy for allocation of patients to CD and UCD for RASi only, RASi + GLP1a, RASi + MCRa, and RASi + SGLT2i provided by Δ_*R*_, Δ_*G*_, Δ_*M*_, and Δ_*S*_, respectively are given in Table 4. These models are used to calculate the expected increase/decrease in ΔeGFR, which can be used to inform the clinician on whether or not to prescribe the add-on drug (Figure 2).

**Table 1:** Expert selection of key continuous predictors.

**Marker**	**Symbol**
Estimated glomerular flow rate	eGFR
Urine albumin-creatinine ratio	UACR
Systolic blood pressure	SBP
Diastolic blood pressure	DBP
Hemoglobin	HB
Serum cholesterol	TOTCHOL
Body mass index	BMI
HbA_1*c*_	HBA1C
Age	Age

**Table 2:** Reduced data set.

**Marker**	**Name**	**Data set**
eGFR	Estimated glomerular filtration rate	Expert
DPP4	Dipeptidyl peptidase-4	Extended
ICAM1	Intercellular Adhesion Molecule 1	Extended
LEP	Leptin	Extended
AGE	Age	Expert
ADIPOQ	Adiponectin	Extended
TOTCHOL	Total serum cholesterol	Expert
SBP	Systolic blood pressure	Expert
SERPINE1	Plasminogen activator inhibitor-1	Extended

The PROVALID identifiers are displayed in the left column. The data sources are given in the far right column. The labels “Expert” are from the expert data set. Those labeled “Extended” are from the PROVALID dataset that are not also in the expert set.

**Table 3:** Top five biomarkers for each treatment population.

**RASi alone**	**RASi + GLP1a**	**RASi + MCRa**	**RASi + SGLT2i**
eGFR	LEP	DPP4	LEP
DPP4	DPP4	LEP	SERPINE1
ICAM1	eGFR	eGFR	DPP4
LEP	ICAM1	TOTCHOL	eGFR
AGE	ADIPOQ	ICAM1	TOTCHOL

The columns represent the variables, in descending rank order, that are important for each treatment model. For instance, in the model for the population that took GLP1a as an add-on treatment, LEP had the greatest effect on the prediction.

**Table 4:** Diagnostics of model quality for four treatment models.

**Model**	**Output**	**ACC**	**SE**	**SP**	**# patients**
RASi alone	Δ_*R*_	0.78	0.50	0.93	277
RASi + GLP1a	Δ_*G*_	0.92	0.93	0.92	52
RASi + MCRa	Δ_*M*_	0.87	0.77	0.92	64
RASi + SGLT2i	Δ_*S*_	0.93	0.81	0.97	104

The predictions of Δ*e*GFR for RASi alone, RASi + GLP1a, RASi + MCRa, and RASi + SGLT2i are given by Δ_*R*_, Δ_*G*_, Δ_*M*_, and Δ_*S*_, respectively. The quantity Δ_*R*_ is the composite biomarker. The quantities Δ_*G*_, Δ_*M*_, and Δ_*S*_ are the model outputs for RASi plus GLP1a, RASi plus MCRa, and RASi plus SGLT2i, respectively. The data are divided into UCD and CD. Model output predictions were performed using leave-one-out validation performed on the entire population for each treatment. Sensitivity (SE) is the fraction of observed UCD patients that were predicted correctly. Specificity (SP) is the fraction of observed CD patients that were predicted correctly. Accuracy (ACC) is the total number of patients that were predicted correctly. The number # of patients in each population is displayed in the last column. As explained in the Appendix, the models contain random number generators, which causes slight run-to-run variation in outputs for the same data set.

## 4. Discussion

In this study, we identified a composite biomarker panel that predicts the annual change in ΔeGFR for four different drug treatments. The baseline treatment was the blockage of the renin angiotensin system by ACEi or ARB therapy. Next, three additional drugs on top of RASi, SGLT2i, MCRa, or GLP1a, were tested. Only one add-on drug at a time was allowed on top of RASi treatment in our dataset. We do not have data on multi-drug combination therapies. Higher estimated increase of eGFR for add-on patients compared with RASi-alone patients indicates that prognosis is improved with the add-on treatment. The personalized biomarker value thus identifies which patients may benefit from the additional treatment.

Several studies have addressed mid- to long-term prognostic and predictive biomarkers in DKD and found reasonable discrimination on a cohort level. However, for individuals with their sensitivity and specificity is modest at its best (24) at least partially due to longitudinal intra-individual variability in progression. Consequently, our approach relies on short-term prediction and a direct comparison of published mid- to long-term markers with our composite short-term biomarker is not adequate.

An added value of the composite biomarker is that it increases the resolution of biomarkers to identify patients that respond differently to treatments. This can be seen in Figures 1, 2, where the composite biomarker identifies a specific cluster of low-Δ_*R*_ patients that respond positively to add-on treatments.

The study was motivated by the need for precision treatment for DKD. Precision drug therapy is becoming increasingly important in this area as more and more options to intervene become available. Several efforts to predict inter-individual differences in kidney disease progression to “hard” long-term outcome endpoints (incidence of e.g., end-stage kidney disease) under specific therapies have already been undertaken. While the identified markers/ or marker panels show some promise in cohorts, their accuracy at the level of an individual is modest, limiting their value in bedside medicine. One reason for this shortcoming is the fact that progression of chronic kidney disease (e.g., as assessed by a decrease in eGFR) also shows considerable variability within an individual over time. Even under stable drug therapy, periods of falling eGFR can be followed by recovery under stable treatment (21). Our study design respects this aspect by restricting predictions to relatively short (annual) intervals of follow-up. Of note, this approach closely follows current guidelines that recommend repetitive annual assessment of eGFR to adjust the treatment strategy (25). Clearly, the magnitude of change in eGFR to detect is smaller with shorter follow-up and a “misclassification” based on spontaneous eGFR variability must be taken into account. Our discriminatory threshold for the definition of CD and UCD takes these caveats also into consideration.

The modeling process was constrained by the large number of possible inputs and a small amount of available data in relation to the number of possible inputs. To address these constraints, we reduced the number of variables by identifying those variables that had the biggest effect on the output ΔeGFR. We also removed collinear variables that contained redundant information. We used PLS, which is a linear process, to achieve both these goals (Appendix). We also addressed the constraint that the chosen reduced set of variables must include inputs that are measurable in a clinical setting and that are reasonably familiar to clinicians. To do this, we heuristically ran both data sets generated by experts and the complete PROVALID data set through PLS pre-selection. We then combined the highest ranking inputs from both data sets into a single reduced data set (Table 2). We then used the reduced data set including the PLS regression output as inputs to a non-linear neural network model that is designed to extract information from small amounts of data as can be found in control problems (26). Managing disease treatments is a biological control problem. The output from the neural network is the composite biomarker Δ_*R*_ used to identify optimal treatment regimes. This is done by comparing observed and predicted outcomes from various treatments with each other.

The most immediate use of the composite biomarker is to inform the clinician on the predicted change of ΔeGFR if a treatment with an add-on drugs is applied. The model possibly may be used to access the particular disease pathway in each patient. For instance, the data and model predictions of Figures 1, 2 indicate that add-on treatments may be preventing outcome degradation for sicker patients with lower values of the composite biomarker Δ_*R*_. An examination of the fundamental biomarkers for these low-Δ_*R*_ patients may indicate the mechanism that prevents this degradation in outcome. This is currently under investigation. This information may identify additional drug targets.

The biomarkers were identified by an heuristic approach on a set of continuous variables and without consideration of proteomics. A more exhaustive inclusion practice (e.g., by mining urinary proteomics data) may very well identify other characteristics that add to or substitute for components. On the other hand, the markers finally entering the algorithm ideally are reasonably accessible and allow pathophysiological interpretation as this increases acceptance of healthcare providers, payers, and physicians. Another weakness of our current model is that we were restricted to continuous markers as inputs, leaving out discrete and binary variables. We are currently exploring the possibilities of including them as well. Finally, the model was built and validated on the PROVALID data set. Other data sets are in preparation for external validation. The basic principle of our approach is that longitudinal intra-individual variability in progression decreases the accuracy of any prediction marker with extended follow-up periods. This will be the topic of a follow-on study. However, one specific property of the model is that it predicts the change in eGFR 1 year in advance. It is possible to extend the model to predict multiple years in advance; thus, it is possible to extend the model to predict multiple repetitive years. This will require that the model also predicts all fundamental biomarkers 1 year in advance, rather than just the output ΔeGFR. This will require that the model predict all fundamental biomarkers 1 year in advance, rather than just the output ΔeGFR. The prediction can then be iterated to provide predictions multiple years in advance. This process is under study.

In summary, this study identifies a composite biomarker for DKD that is an aggregate of fundamental biomarkers easily accessible to clinicians. The composite biomarker can be used to inform the decision to maintain a patient on a RASi-only treatment or to add GLP1a, MCRa, or SGLT2i to the RASi treatment. Clinical access to this model and related models is currently being developed, tested, and prepared for the approval process.

## Data availability statement

The data analyzed in this study is subject to the following licenses/restrictions: data owned by the European Union DC-ren project. Requests to access these datasets should be directed to GM, gert.mayer@i-med.ac.at.

## Ethics statement

The studies involving human participants were reviewed and approved by the Ethics Committee of the Medical University Innsbruck; DC-ren approval number: EK Nr:1188/2020, date 19.06.2020. The DC-ren cohort consists of patients from PROVALID and written informed consent to participate in this study was obtained from all patients.

The PROVALID dataset used in this study was approved by the local Institutional Review Board (IRB) in each participating country, and are listed below. Signing an informed consent was a prerequisite for study participation in all countries.

Austria: Ethical approval from the Ethics Committee of the Medical University Innsbruck AN4959 322/4.5370/5.9 (4012a); 29.01.2013 and approval of the Ethics Committee of Upper Austria, Study Nr. I-1-11; 30.12.2010. Hungary: Approval from Semmelweis University, Regional and Institutional Committee Of Science And Research Ethics; No.12656-0/2011-EKU (421/PV11.);17.06.2011. United Kingdom: Approval from WoSRES, NHS; Rec. Reference:12/WS/0005 (13.01.2012). Netherlands: Approval of the Medical Ethical Committee of the University Medical Center Groningen, ABRnr. NL35350.042.11. Poland: Approval from Ethics Committee of the Medical University of Silesia, KNW/022/KB1/78/11/, date 07.06.2011.

## Author contributions

RJ: Conceptualization, Formal analysis, Investigation, Methodology, Software, Validation, Visualization, Writing—original draft, review, and editing. SA: Writing—review and editing. VD: Writing—review and editing. GM: Conceptualization, Funding acquisition, Project administration, Supervision, Writing—original draft, review, and editing. IP: Conceptualization, Formal analysis, Funding acquisition, Investigation, Project administration, Supervision, Validation, Writing—review and editing. CS: Data curation, Writing—review and editing. DS: Writing—review and editing.

## Figures and Tables

**Figure A1:**
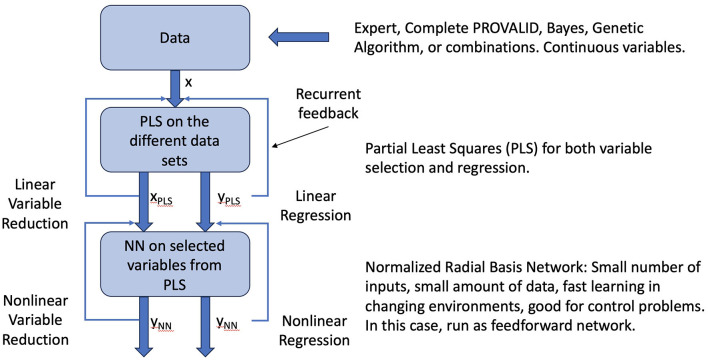
Raw data can come from experts, the PROVALID data set, or other preproccessed sources. Preprocessing might include dimensionality reduction with Bayesian networks, genetic algorithms, or other dimensionality reduction schemes as indicated in the figure. The data are run through PLS to create a reduced vector of inputs ranked by how they affect the output and to create a linear estimate of the output. These are fed back into the PLS process and iterated to convergence. The converged outputs are inputs to the normalized radial basis network. The output is both a non-linear estimate of the output and a non-linear reduction of the input variables. The network is iterated to convergence yielding the final output Δ_*R*_.

## Appendix: The hybrid machine-learning approach

### PLS

The simplest model we consider to predict and maximize Δ*e*GFR is a linear model.

(A1)
$$\begin{array}{l} ŷ=B^{T}x_{b}, \end{array}$$

where *x*_*b*_ is a vector of length p of biomarker values, ŷ is a the estimate of the value of Δ*e*GFR, and B is a vector of length p of coefficients. The coefficients *B* can be determined by LR as suggested in (22). Here, we calculate *B* with PLS. PLS has an advantage over LR by reducing the issue of colinearity. PLS also can be used to rank the importance of the input biomarkers in determining the output ŷ. Therefore, not only can PLS be used in estimating the output but it can also be used in input dimensionality reduction, reducing the value of *p*. We use both of these features of PLS in this study. PLS is available as a package in python and MATLAB. Here, we use plsregres from the MATLAB library. Note that PLS uses a random-number generator and, therefore, has slight variation in the output from run to run even for the same data set.

Models can be divided into two classes, feedforward and recurrent. Feedforward models map an input smoothly into an output, while recurrent networks map inputs and outputs into outputs. Recurrent models usually require iteration for evaluation. A guess for the output is supplied and an updated value for the guess is calculated by mapping the initial guess and the inputs into the second guess. This process is repeated until the process converges. A simple feedforward model such as Eq. 1 can describe a smooth dependence of ŷ on the biomarkers, but the evolution of DKD can behave discontinuously as a result of vicious cycles in the disease progression. In other words, the disease progress can increase suddenly over a period of months, which is shorter than the time between annual visits. This appears as a discontinuity in the data.

Recurrent models are able to model discontinuities [Sec. 1.3](26). Specifically, we model discontinuous jumps with a polynomial in the output ŷ. For the case of a cubic polynomial, Eq. 1 becomes

(A2)
$$\begin{array}{l} a_{3}{ŷ}^{3}+a_{2}{ŷ}^{2}+ŷ=B^{T}x_{b}, \end{array}$$

where *a*_2_ and *a*_3_ are coefficients to be determined. The output ŷ can be evaluated iteratively. Equation 2 can be written

(A3)
$$\begin{array}{l} ŷ=f\left(x_{b},ŷ\right), \end{array}$$

where

(A4)
$$\begin{array}{l} f\left(x_{b},ŷ\right)=B^{T}x_{b}-a_{2}{ŷ}^{2}-a_{3}{ŷ}^{3}, \end{array}$$

which is non-linear in ŷ but linear in coefficients *B*^*T*^, *a*_2_, and *a*_3_. Equations 3, 4 describe a recurrent model that models a cubic equation. The particular case of a cubic model is described in [Sec. 1.3](26). The output is evaluated by making an initial guess ŷ_0_ for ŷ on the RHS of Eq. 3 and using *f* to update the guess. The process is a one-dimensional map of ŷ onto itself. The process is repeated until the difference between successive guesses becomes smaller than a threshold value. Here, we find that the differences become smaller than 1% after 10 iterations. The guesses converge to one of as many as three possible real fixed points.

The final fixed point is determined by two factors, the initial guess and the slope:

(A5)
$$\begin{array}{l} f^{\prime }=\frac{\partial f}{\partial ŷ} \end{array}$$

at each of the fixed points. It is easy to show that a stable fixed point obeys the condition

(A6)
$$\begin{array}{l} |f^{\prime }|<1 \end{array}$$

In other words, if a fixed point violates Eq. 6 at the fixed point, then that point is not stable. The system will avoid that fixed point and converge to one in which Eq. 6 is satisfied. If the slope approaches zero, the system converges or diverges from the fixed point very slowly. We have not seen this situation in practice. The final fixed point is not only a fixed point that is stable but also one for which the initial guess lies within the fixed point's basin of attraction. The details of the boundaries for the basins of attraction are determined by $B^{T}x_{b}$. The classic reference on the topic of fixed points and basins of attraction for one-dimensional non-linear dynamics is Feigenbaum (27).

The polynomial model, Eqs. 2, 4, can fit into the PLS framework. In order to train the coefficients, the actual output *y*^*^ is substituted for the estimated output ŷ in Eq. 4. The inputs *x* to PLS are a vector of length *p*+2.

(A7)
$$\begin{array}{l} x=\left[x_{b},-{\left(y^{*}\right)}^{2},-{\left(y^{*}\right)}^{3}\right] \end{array}$$

The fixed points, therefore, are trained on actual outputs *y*^*^, and we expect the iterative evaluation process to converge to an estimate for the actual output value *y*^*^. The coefficients *B*, *a*_2_, and *a*_3_ are given by the PLS process. Here, we choose the initial guess for ŷ to be ŷ_0_ = 0. The consequences of this choice are tested numerically.

### Biomarker selection

Biomarkers were selected from two data sets, the expert data set (Table 2) (22) and the complete PROVALID data set, which also contains the expert data set as a subset. Only continuous variables were considered. The number of PLS components was chosen to be five. The most important variables in both the expert and the PROVALID data sets were selected and combined into a single data set (Table 2). This combined data set was used for all subsequent modeling.

### PLSNN

Neural networks are commonly used for modeling data. The most common networks used for deep learning, however, require massive amounts of data to train (28). In this study, we use a class of localized networks (29) that can provide quick and accurate results on small problems such as those we have in this study (see Figure A1). Normalized radial basis function networks (NRBNs) have demonstrated good utility in control problems that require a small number of inputs and that live in a changeable environment (26, 30–34).

In order to increase the model accuracy beyond that achievable by PLS, we input the variables, coefficients, and output from PLS into NRBN to obtain an improved estimate for *y*^*^. A training set of sample observables is used to fit trainable parameters. The combined model is designated PLSNN.

The architecture of PLSNN is (26, 30)

(A8)
$$\hat{\hat{y}}=\sum_{i=1}^{q}\sum_{j=1}^{p}a_{ij}B_{j}x_{j}\;u[d(x^{\prime },x_{i}^{\prime }],$$

where $\hat{ŷ}$ is the PLSNN estimate, *x* is the expanded input vector given by Eq. 7, *B* are the corresponding PLS coefficients, *u* is a normalized basis functions, the hyperparameter *q* is the number of basis functions, *x*′ is a selected set of inputs that taken from PLS outputs, $x_{i}^{\prime }$ are the basis function centers chosen randomly from the training set, $d\left(x^{\prime },x_{i}^{\prime }\right)$ is a dimensionless distance measure between *x*′ and $x_{i}^{\prime }$, and *a*_*ij*_ is a set of trainable parameters. Here, the two-dimensional input vectors *x*′ are given by

(A9)
$$\begin{array}{l} x^{\prime }\to \left[y^{*},ŷ\right] \end{array}$$

for training the network and

(A10)
$$\begin{array}{l} x^{\prime }\to \left[\hat{ŷ},ŷ\right] \end{array}$$

for evaluation. The basis centers $x_{i}^{\prime }$ are q random selections from the training vectors given in Eq. 9. Note that this randomness causes small variation in the output from run to run for the same data set. In other words, the network is trained on actual data, which is available from the training set, but it is evaluated using the iterated process described above because the output is not yet known for the test/evaluation set.

We take the dimensionless distance d between an input vector and a basis center to be the Euclidean distance (29).

(A11)
$$\begin{array}{l} d\left(x^{\prime },x_{i}^{\prime }\right)=\beta \frac{{\left(x^{\prime }-x_{i}^{\prime }\right)}^{T}\left(x^{\prime }-x_{i}^{\prime }\right)}{\sigma^{*}} \end{array}$$

where the hyperparameter β is a scaling parameter and σ^*^ is the standard deviation of all the observed outputs *y*^*^ in the training set. We typically set β = 1. Comparable results are found for values β = 0.2 to 5.

The normalized basis functions *u* are given by

(A12)
$$u[d(\hat{x},{\hat{x}}_{i})]=\frac{\rho [d(\hat{x},{\hat{x}}_{i})]}{\sum_{i=1}^{q}\rho [d(\hat{x},{\hat{x}}_{i})]}$$

where we used the convenient choice for the localized basis function (29)

(A13)
$$\begin{array}{l} \rho \left(d\right)=\exp\left(-d\right) \end{array}$$

For evaluation, $\hat{ŷ}$ is an estimate for *y*^*^. The PLSNN estimate for $\hat{ŷ}$ is evaluated in the same iterative manner as the PLS estimate, Eq. 3. The initial guess for $\hat{ŷ}$ is the PLS estimate $\hat{ŷ}=ŷ$.

We train the network with the projection-operator technique [see, for example, (26, 30)]:

(A14)
$$\begin{array}{l} \Delta a_{ij}=\nu [y^{*}-\hat{\hat{y}}]\frac{v_{ij}}{\sum_{i=1}^{q}\sum_{j=1}^{p}v_{ij}^{2}}. \end{array}$$

where the hyperparameter 0 < ν < 1 is the learning rate and

(A15)
$$\begin{array}{l} v_{ij}=B_{j}x\;u\left[d\left(x^{\prime },x_{i}^{\prime }\right)\right]. \end{array}$$

The outcomes are insensitive to the learning rate. We take the rate to be ν = 0.1. Equation 14 is updated for every sample *x*, *x*′, and $\hat{ŷ}$ in the training set. Multiple training sets were formed by removing one sample for testing, shuffling the samples used as basis centers, and shuffling the order in which the samples were presented to the training algorithm Eq. 14.

A test set was created by randomly selecting and removing one sample from each training set. PLSNN was not trained on this sample. This sample formed a member of the leave-one-out test set. Performance parameters were calculated from this test set.

We built four separate models of the annual percent change (Δ*e*GFR), one for each sub-population that receives a particular drug treatment (Table 4). The baseline sub-population are those patients who only received renin-angiotensin system inhibitor (RASi) (35). The remaining three treatment regimes involved the addition of a second drug to the baseline treatment: sodium-glucose cotransporter-2 inhibitor (SGLT2i) (36), glucagon-like peptide-1 (GLP-1a) (37), and antimineralocorticoid receptor antagonist (MCRa) (38). Distinct variable selection and Δ*e*GFR prediction was performed for each of the four models.

The composite biomarker Δ_*R*_ is the estimated value of Δ*e*GFR for the RASi-Alone Model.
