# Supplementary material for: Candidate composite biomarker to inform drug treatments for diabetic kidney disease
Source: Front Med (Lausanne). 2023 Nov 1;10:1271407. doi: 10.3389/fmed.2023.1271407 (PMC10646536; doi:10.3389/fmed.2023.1271407)
Supplement: Supplementary file 1 [file Data_Sheet_1.pdf]

Supplementary Material

SUPPLEMENT

Supplementary Table 1

Symbol	Name	ENSG	sample type
AGER	advanced glycosylation end-product specific receptor	ENSG00000204305	blood
IL18	interleukin 18	ENSG00000150782	blood
TNFRSF1A	TNF receptor superfamily member 1A	ENSG00000067182	blood
MMP7	matrix metallopeptidase 7	ENSG00000137673	blood
IL6	interleukin 6	ENSG00000136244	blood
TNF	tumor necrosis factor	ENSG00000232810	blood
ICAM1	intercellular adhesion molecule 1	ENSG00000090339	blood
VEGFA	vascular endothelial growth factor A	ENSG00000112715	blood
LEP	Leptin	ENSG00000174697	blood
ADIPOQ	adiponectin, C1Q and collagen domain containing	ENSG00000181092	blood
SERPINE1	serpin family E member 1	ENSG00000106366	blood
DPP4	dipeptidyl peptidase 4	ENSG00000197635	blood
LGALS3	galectin 3	ENSG00000131981	blood
CST3	cystatin C	ENSG00000101439	blood
NPHS1	NPHS1 adhesion molecule, nephrin	ENSG00000161270	urine
MMP9	matrix metallopeptidase 9	ENSG00000100985	urine
LCN2	lipocalin 2	ENSG00000148346	urine
FGF21	fibroblast growth factor 21	ENSG00000105550	urine
THBS1	thrombospondin 1	ENSG00000137801	urine
HAVCR1	hepatitis A virus cellular receptor 1	ENSG00000113249	urine
MMP2	matrix metallopeptidase 2	ENSG00000087245	urine
CCL2	C-C motif chemokine ligand 2	ENSG00000108691	urine
EGF	epidermal growth factor	ENSG00000138798	urine
SIRT1	sirtuin 1	ENSG00000096717	blood
NPPB	natriuretic peptide B	ENSG00000120937	blood

Supplementary Table 2

	RASi only group %, Mean ± SD or Median and IQR as appropriate	RASI plus SGLT2-I group %, Mean ± SD or Median and IQR as appropriate	RASi plus MCRa group %, Mean ± SD or Median and IQR as appropriate
Gender female [%]	50,3	31,3	35,3
Smoking status ever/current [%]	45,0	46,9	41,2
Age [years]	65,4 ± 8,9	60,3 ± 9,6	68,2 ± 8,2
BMI [kg /m^2]	32,2 ± 5,5	32,7 ± 6,0	32,8 ± 5,7
Diabetes duration [years]	13,6 ± 7,7	14,7 ± 7,5	13,4 ± 9,5
Hypertension [%]	90,5	87,5	79,5
Hypertension duration [years]	16,2 ± 10,7	14,7 ± 8,8	24,1 ± 13,8
MDRD eGFR [ml/min/1.73 m^2]	64,9 ± 15,6	71,5 ± 13,1	63,3 ± 15,2
Urinary albumin excretion [mg/g Creatinine]	10,2 (4,5 - 29,9)	13,7 (3,7 - 33,0)	11,9 (4,3 - 35,9)
Systolic blood pressure [mmHg]	137,4 ± 15,3	132,9 ± 15,1	136,9 ± 18,4
Diastolic blood pressure [mmHg]	78,8 ± 9,6	78,9 ± 7,4	79,0 ± 9,9
HbA1c [%]	7,5 ± 1,4	8,1 ± 1,5	6,9 ± 1,1
CRP [mg/dl]	0,7 ± 1,6	0,5 ± 1,0	0,6 ± 0,6
Serum albumin [g/dl]	4,5 ± 0,4	4,7 ± 0,4	4,5 ± 0,5
Hemoglobin [g/dl]	13,8 ± 1,5	14,7 ± 1,4	13,7 ± 2,0
LDL cholesterol [mg/dl]	96,9 ± 35,9	92,4 ± 35,8	79,1 ± 23,9
HDL cholesterol [mg/dl]	48,6 ± 13,9	44,6 ± 10,5	45,4 ± 13,7
Serum triglycerides [mg/dl]	181,5 ± 120,5	255,5 ± 310,8	173,3 ± 78,2
Serum potassium [mmol/l]	4,5 ± 0,5	4,4 ± 0,4	4,4 ± 0,5
Serum phosphate [mmol/l]	1,1 ± 0,2	1,2 ± 0,1	1,1 ± 0,2
Serum calcium [mmol/l]	2,4 ± 0,1	2,4 ± 0,1	2.4 ± 0,1
Serum uric acid [mg/dl]	5,5 ± 1,4	4,8 ± 1,2	5,8 ± 1,7
Sulfonylureas [%]	36,2	34,1	32,4
Glinides [%]	29,4	39,0	8,1
DPPIV inhibitors [%]	0,3	2,4	2,7
Thiazolinediones [%]	2,5	4,9	2,7
Metformin [%]	73,6	85,4	73,0
Insulines [%]	36,2	36,6	48,6
